# Computation screening for incorrectly determined cocrystal structures

**DOI:** 10.1107/S205252062500068X

**Published:** 2025-02-25

**Authors:** Simona Chalupná, Michal Hušák, Jan Čejka, František Fňukal, Jiří Klimeš

**Affiliations:** a Department of Solid State Chemistry University of Chemistry and Technology Prague Technicka 5 Prague 166 28 Czechia; b Department of Chemical Physics and Optics, Faculty of Mathematics and Physics Charles University Ke Karlovu 3 Praha 2 121 16 Czechia; Academy of Sciences of the Czech Republic, Czechia

**Keywords:** DFT-D, salt, cocrystal, cocrystal structures, verification

## Abstract

Pharmaceutical solid forms, like salts and cocrystals, play a crucial role in drug formulation. Despite differing mainly by a single hydrogen atom, the regulatory requirements set by the US Food and Drug Administration for these forms vary significantly. We previously developed a DFT-based computational method to distinguish salts from cocrystals. This method, validated on 95 structures, performed well for systems where hydrogen bonds were longer than 2.613 (16) Å. Here, benefits of the rSCAN functional over the PBE functional are discussed. We expand the dataset to 404 cocrystal models. Analysis confirms that 301 of these forms are indeed cocrystals. Additionally, 87 salt–cocrystal continuum forms are identified and 16 cocrystals are classified as possible salts. These 16 problematic structures are further investigated and for seven of them, single crystals were grown and their structure determined using single-crystal X-ray diffraction. Among the phases exhibiting salt-like behaviour, five of them are identified as salts. In some cases, rSCAN alone gives unreliable results for strong hydrogen bonds, but these discrepancies are often corrected using better-renormalized or hybrid functionals (*i.e.* r2SCAN, PBE0 and PBE50). For future calculations, we recommend using the r2SCAN functional for salt–cocrystal differentiation, as it provides reliable results for O—H⋯N bonds longer than 2.554 (5) Å. The r2SCAN functional offers a good balance between accuracy and computational efficiency for systems with longer O—H⋯N bonds.

## Abstract 2

Computational salt–cocrystal differentiation, based on density functional theory improved by up-to-date functionals rSCAN and r2SCAN, has been tested for 404 structures. Several problematic structures have been redetermined.

## 1. Introduction

There is a broad range of solid forms available for pharmaceutical molecules. A cocrystal is a stoichiometric multicomponent host:guest compound formed from two or more components that are solid in the pure state and at room temperature (Aakeröy & Salmon, 2005 ▸; Aitipamula *et al.*, 2012 ▸). Cocrystals are rapidly evolving as a prominent type of solid pharmaceutical form (Aitipamula *et al.*, 2012 ▸). Pharmaceutical salt is an ionisable drug that has been combined with a counter-ion to form a neutral complex. About half of all pharmaceuticals available in the market are in salt form (Gupta *et al.*, 2018 ▸; Aitipamula *et al.*, 2012 ▸). The difference between a salt and a cocrystal (for molecules where hydrogen transfer is responsible for the ionization state) is given only by the position of a single hydrogen atom. As this difference is quite small, it is essential to develop new techniques that can accurately identify the precise location of the relevant hydrogen atom. Differentiation of salt from cocrystal compounds holds significant importance within the pharmaceutical industry, both for regulatory purposes and overall quality control. The US Food and Drug Administration (FDA) has explicitly outlined the necessity for the accurate identification of a pharmaceutical phase in its 2018 guidelines (FDA, 2018 ▸).

Numerous well-established techniques for the differentiation between salt and cocrystal already exist. Among these, single-crystal X-ray structure determination is the most commonly used method. Methods such as X-ray photoelectron spectroscopy, solid-state nuclear magnetic resonance or calculating Δp*K*_a_ are also available. The most precise method for locating H atoms is neutron diffraction, which unfortunately requires large single crystals and an expensive neutron radiation source. However, each of these methods has its limits and cannot be applied to all systems (Jeffrey, 1997 ▸; Nygren *et al.*, 2005 ▸; Stevens *et al.*, 2010 ▸; Gardiennet-Doucet *et al.*, 2006 ▸; Cruz-Cabeza, 2012 ▸).

We have already developed and partially tested a computational method for salt cocrystal differentiation based on density functional theory (DFT) energy calculation (Hušák *et al.*, 2022 ▸). We improved this method in the current study by applying the conclusions from Hušák *et al.* (2022 ▸) and testing a larger number of structures. The key to improvement was the use of the most up-to-date meta-GGA rSCAN functional instead of the PBE one. The use of the rSCAN functional does not significantly increase computational cost.

This study aimed to extend the number of experimental crystal structures on which the method was tested and prove that the rSCAN functional could give better results than the previously used PBE functional. Another goal was to identify incorrectly determined cocrystal structures.

## 2. Methods

### 2.1. Computational screening

The data source for testing the DFT method was the Cambridge Structural Database (CSD) (Groom *et al.*, 2016 ▸). We tested structures from a cocrystal deposition code list from the zone (−1 ≤ Δp*K*_a_ ≤ 4), as defined in the article ‘Acid–base crystalline complexes and the p*K*_a_ rule’ (Cruz-Cabeza, 2012 ▸). Calculations were performed on 404 of the 495 cocrystals in the list. This is an expansion from the 95 cocrystals used in our previous work (Hušák *et al.*, 2022 ▸). The current study covers structures with hydrogen atoms not explicitly determined from the difference Fourier map and structures with any *R*-factor value. Out of the 495 cocrystal structures, 91 were rejected because of disorder, nonsensical structures (missing or extra atoms, wrong geometry) or when it was not clear which hydrogen atom was responsible for the transition between the salt and the cocrystal (large systems with a high number of hydrogen bonds). We rejected disordered structures from our study because they are difficult to model with DFT. There are methods to model disorder using DFT [such as the Virtual Crystal Approximation (VCA) or by using supercells], but these have not been extensively tested for such complex calculations as those presented in our article.

The first step of the process was to identify the critical hydrogen atom responsible for the transition between the cocrystal and the salt. Subsequently, a structure model opposite the original was prepared (*i.e.* false salt was prepared from the cocrystal by shifting the hydrogen atom from donor to acceptor). The artificial incorrect hydrogen atom position was created by placing the hydrogen on the donor–acceptor line at a distance of 0.95 Å from the incorrect atom. The position of all atoms in the studied crystal structures was then geometry optimised. When the optimisation led to the same state as the experimental one, the structure was considered to be correctly solved. During the main screening using the rSCAN functional, geometric optimisation was performed on 404 cocrystals from the Δp*K*_a_ zone (−1 ≤ Δp*K*_a_ ≤ 4). Optimisation was always performed from both artificial salt and cocrystal starting models. A schematic diagram of this workflow can be found in Hušák *et al.* (2022 ▸). These models were prepared using *checkCIF-DFT* software (Fňukal, 2022 ▸). With this software, a hydrogen atom can easily be shifted to an opposite artificial position. The *checkCIF-DFT* software is also capable of preparing the necessary input files for different quantum mechanics software (including the used *CASTEP* code) and preparing setup files for an automatic run on supercomputers.

In addition, calculations were performed from the starting model of the cocrystal, that is from the original structure without any modifications. Such calculations are important for structures with dual energetic minima for both salt and cocrystal. In a situation in which the calculation starting from salt converges into salt, we must check whether the predicted result is not pure salt.

The DFT optimisation of atomic positions was performed using the *CASTEP* code (Clark *et al.*, 2005 ▸). We used fixed unit-cell parameters because this compensates for DFT Pulay stress errors related to lattice calculation, temperature effects and situations where there are voids in the structure.

We used the rSCAN functional with many-body dispersion (MBD) correction and fine basis precision (Bartók & Yates, 2019 ▸; Hermann & Tkatchenko, 2018 ▸). The complete set of parameters for the calculations setup is given in Section S2 in supporting information. Computation was performed on the Karolina supercomputer at TU Ostrava, Czech Republic.

During the screening, we identified 16 problematic structures. These structures experimentally determined as cocrystal always converged into salt form from both the artificial salt model and the original experimentally determined cocrystal form. For four of these 16 problematic structures, results violated the methodology reliability rules determined in our previous article. The experiment–calculation discrepancy can be related to the issue with the DFT functional used. The rSCAN functional used falls into the meta-GGA category. This functional suffers from a so-called self-interaction error (Bursch *et al.*, 2022 ▸). This can be reduced using hybrid functionals (*i.e.* PBE0, PBE50). We also tested the PBE0 functional and the PBE50 functional in problematic cases as well. As the PBE0 functional contains 25% Hartree–Fock exact exchange energy, it should reflect H bonds exactly. The PBE50 functional contains 50% Hartree–Fock exact exchange energy so it should prefer cocrystal over salt even more than PBE0. The influence of Hartree–Fock exact exchange energy on the salt/cocrystal ratio determination was demonstrated by Bal & Collas (2024 ▸) with the same conclusion (more Hartree–Fock exact exchange energy shifts toward cocrystal). In addition to the hybrid functional usage, we tested the r2SCAN functional with MBD dispersion correction as a less computationally expensive alternative but more numerically precise than rSCAN in problematic cases (Furness *et al.*, 2020 ▸).

### 2.2. Crystallization and X-ray diffraction

For structures with no experimental data available or missing low-temperature high-resolution data, we tried to reproduce the phase crystallisation and perform structure redetermination ourselves. Crystallisation was feasible and successful for seven structures (more details in Section 3); the crystallisation conditions are described in Section S3 in supporting information). The structural data from single crystals were collected using a Bruker D8 VENTURE system. The diffractometer was equipped with a Photon II 7 CPAD detector, a multilayer monochromator and a Mo *K*α1 (λ = 0.71073 Å) sealed tube. The crystal structure data were measured at 180 K. Data reduction and absorption correction were performed using *APEX3* software.

### 2.3. Refinement and re-refinement

To obtain the best possible hydrogen atom position, we used the Hirshfeld atom refinement (HAR) method for refinement as implemented in *Olex2* software and the *NoSpherA2* module (Jayatilaka & Dittrich, 2008 ▸; Capelli *et al.*, 2014 ▸; Kleemiss *et al.*, 2021 ▸; Midgley *et al.*, 2021 ▸). In Štoček *et al.* (2024 ▸), we demonstrate that the results obtained from HAR refinement show better agreement with ssNMR data and with advanced quantum mechanical calculation [quantum dynamic with nuclear quantum effects (NQEs) inclusion] compared to the independent atom model (IAM) model in distinguishing between salt and cocrystal forms. Thus, we believe that HAR refinement provides a more accurate characterization of the phase. Additionally, as shown by Štoček *et al.* (2024 ▸), the salt–cocrystal continuum model can be satisfactorily described by hydrogen atoms refined anisotropically using the HAR method. For the HAR wavefunction calculation, we used the Def2-TZVP localised base, r2SCAN functional and *Orca 5.0* software (Neese, 2022 ▸). In all cases, refinement was performed using two methods. The first method was based on the refinement of the problematic hydrogen atom in a single position. The second method was based on the refinement of this hydrogen atom as a disordered one. In this case, the donor and acceptor distances to the hydrogen atom were restrained to the value of 0.95 Å, with an s.u. of 0.01 Å. For the final CIF deposition, the refinement results based on the first method were used only because we believe the disorder model does not correctly reflect the real state of the phases. In addition to the crystal structures for which we collected experimental data, using the same process we re-refined the experimental data, available from the original authors, in two cases OGEPIA (Qin *et al.*, 2008 ▸) and TIGNUT (Bu *et al.*, 2007 ▸).

## 3. Results and discussion

The results are summarised in Tables 1 ▸ and 2 ▸. The behaviour of the studied cocrystal structures can be sorted into three groups. The first group contains cocrystals converging into cocrystal from both salt and cocrystal models. The second group contains structures with local energy minima for both cocrystal and salt forms. The third group contains structures experimentally determined as cocrystals but converted into salt from both salt and cocrystal starting models after the rSCAN-functional based geometry optimisation.

### 3.1. Results for structures experimentally determined as cocrystals

A total of 301 cocrystals converged into cocrystals from both the cocrystal and salt starting models. We consider these structures to be experimentally correctly solved. The CSD refcode list of these structures can be found in Section S1.2 in supporting information.

### 3.2. Cocrystals with energy minimum in both cocrystal and salt positions

A total of 87 cocrystals converged into cocrystal from the cocrystal starting model and into salt from salt starting model. The CSD refcode list of these structures is presented in supporting information (Section S1.3). A discussion on the dependence of energy on the hydrogen position for one of these structures (PUJNIS structure and PBE functional) can be found in our previous work (Hušák *et al.*, 2022 ▸). At laboratory temperature, the structures probably coexist in a form of a disordered state between the cocrystal and salt form. The article ‘Importance of Nuclear Quantum Effects for Molecular Cocrystals with Short Hydrogen Bonds’ (Štoček *et al.*, 2022 ▸) describes such systems using Path Integral Molecular Dynamics simulation. The conclusion of this study provided evidence of a temperature-induced hydrogen atom shift in cocrystals with short hydrogen bonds and demonstrated that, for the prediction of the cocrystal/salt solid forms with short hydrogen bond, computations must include NQEs, particularly hydrogen nucleus delocalisation, and temperature effects. The simulation results showed that the hydrogen atom is not precisely located in two positions but is delocalised between the donor and the acceptor of the hydrogen bond. This situation will probably be common for most structures from the salt–cocrystal continuum at room temperature.

### 3.3. Cocrystals for which the rSCAN functional gives energy minimum only in the salt position

**Figure 1:**
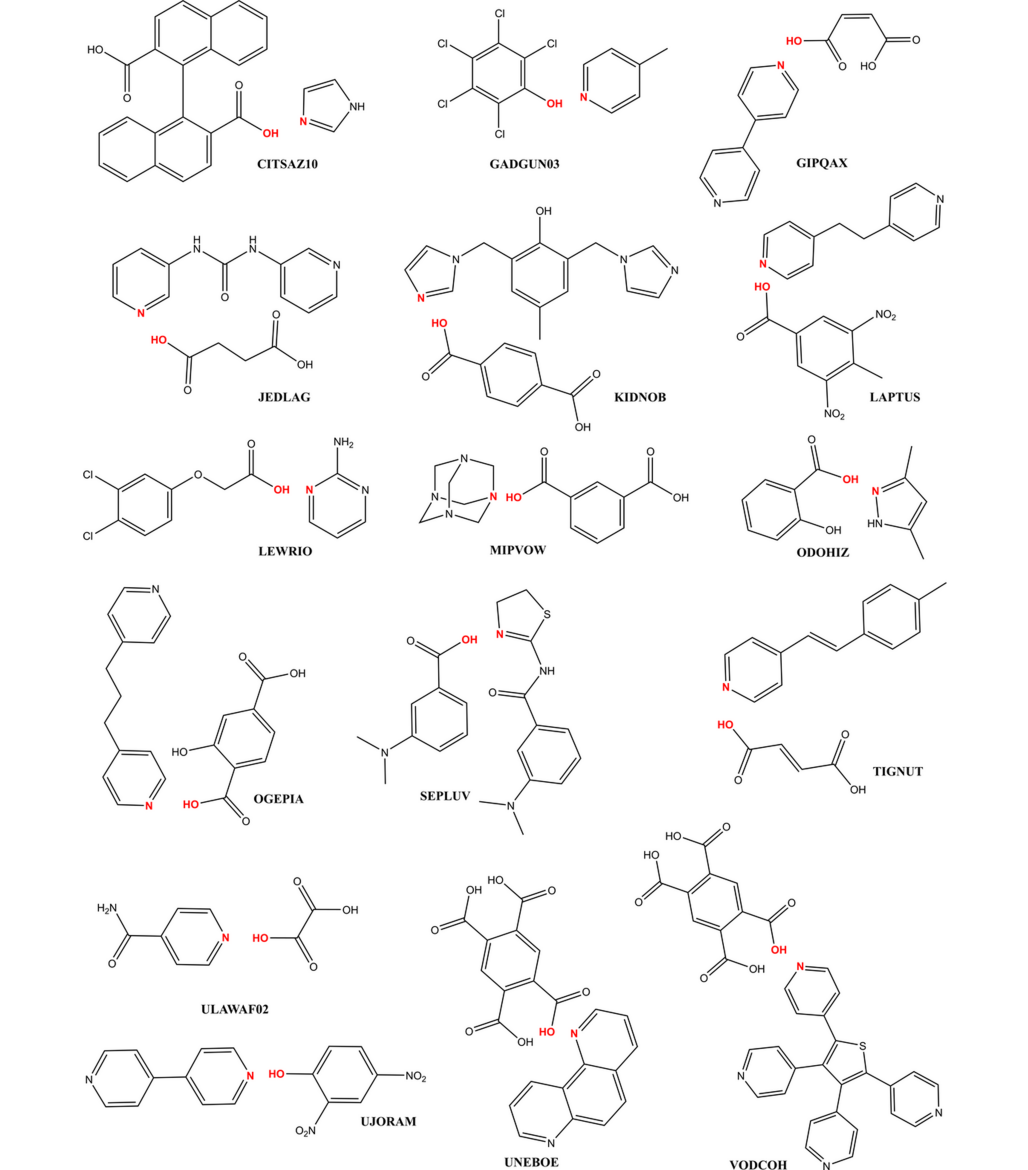
Chemical diagrams of the problematic cocrystals with the highlighted problematic O—H⋯N bonds.

Sixteen structures experimentally determined as cocrystals converged into salt form from both the salt and the original experimentally determined cocrystal form (Table 2 ▸, Fig. 1 ▸). For these structures, we tried to experimentally confirm whether the structure determination was incorrect or to detect the source of the problem with the DFT calculation. For all cocrystals in the problematic category, salt was formed by a transfer from the O—H group donor to the N acceptor. No problems were observed for the other types of potential H transfers in the studied structures. We believe there is a correlation between the O donor to N acceptor distance (reflecting the hydrogen-bond strength) and the reliability of the rSCAN cocrystal/salt prediction, as we already detected in our previous study for the PBE functional. All problematic structures and their analyses are described in individual chapters below. The structures are sorted according to the length of the experimental hydrogen bond (determined by the original authors) from shortest to longest. Twelve of the problematic structures had a strong hydrogen bond shorter than the 2.613 (16) Å (O—H⋯N based) reliability limit determined in our previous study on the PBE functional. The last four (*i.e.* VODCOH, MIPVOW, GIPQAX and CITSAZ10) had the experimental bond longer than the mentioned reliability limit but in the original experiment were determined as cocrystals.

#### 3.3.1. LAPTUS [Bis(3,5-di­nitro-4-methyl­benzoic acid) 1,2-bis­(4-pyridyl)­ethane]

We did not attempt to crystallise the LAPTUS structure (Varughese & Pedireddi, 2005 ▸) because of the unavailability of the components. Instead, we performed additional calculation using the r2SCAN functional. The structure converged into salt again. We then used the PBE0 functional. After this calculation, the structure converged into a cocrystal from the cocrystal starting model. For the salt starting model, the structure converged into salt. Given the presence of an extremely short hydrogen bond of 2.510 (2) Å, we considered that the DFT method based on the rSCAN and r2SCAN functionals was not sufficient to describe the structure in this case. The original authors probably overlooked the salt–cocrystal continuum character of the phase as detected by PBE0 functional (in the original publication they did not mention how the responsible hydrogen atom was located).

#### 3.3.2. GADGUN03 (4-Methyl­pyridine penta­chloro­phenol)

We successfully crystallised the GADGUN03 phase (Steiner *et al.*, 2001 ▸), which contains an extremely short hydrogen bond [2.519 (6) Å]. In the original publication, the authors discussed thermally induced proton migration. In the structure, they observed that at low temperatures, the responsible hydrogen atom was closer to nitro­gen, and as the temperature increased up to room temperature, the hydrogen atom moved closer to oxygen. There was also no sign of a phase transition in the structure. We collected the data and refined them using the HAR method, with the hydrogen atoms treated anisotropically. The structure appeared to be a transition salt–cocrystal continuum phase, with the hydrogen atom in the middle of the donor and acceptor [N—H distance of 1.23 (5) Å and O—H distance of 1.30 (5) Å]. In the disorder refinement model, the phase showed a hydrogen atom occupancy of 0.50 (5) : 0.50 (5) (salt state : cocrystal state) in the positions restrained near the donor and acceptor. We believe that the structure at 180 K probably exists in the form of a hydrogen atom placed in the middle between N and O disordered by the NQEs. We performed calculations using the rSCAN, r2SCAN, PBE0 and PBE50 functionals. However, the structure always converged into salt. We think that the DFT method at the given level is not able to describe the structure because of the short hydrogen bond. The description of this structure at 180 K requires quantum dynamic effect modelling with an NQEs inclusion (Štoček *et al.*, 2022 ▸) because the DFT calculations model the structure at 0 K state.

#### 3.3.3. ULAWAF02 [Trans-oxalic acid bis­(isonicotinamide)]

We did not attempt to crystallise the ULAWAF02 structure (Schmidtmann *et al.*, 2007 ▸) because it had already been investigated in 2009 through neutron diffraction (ULAWAF03; Schmidtmann *et al.*, 2009 ▸). The neutron data showed that the structure had a salt–cocrystal continuum characteristic with the hydrogen atom placed between O and N (Schmidtmann *et al.*, 2009 ▸) at 100 K. The length of the experimental hydrogen bond in this structure was 2.5294 (11) Å. Initially, we performed the calculation using the rSCAN functional. The structure converged from both starting models into the salt form. After using the r2SCAN functional, the structure converged into the cocrystal from the cocrystal starting model, while the structure converged to salt for the salt starting model. Therefore, in this case the r2SCAN functional was required to be used to obtain an agreement with the experimental characteristic of the phase (salt–cocrystal continuum).

#### 3.3.4. SEPLUV [*N*-(Thia­zolin-2-yl)-3-(*N*,*N*-di­methyl­amino)­benzamide 3-(*N*,*N*-di­methyl­amino)­benzoic acid]

We did not attempt to crystallise the SEPLUV structure (Lynch *et al.*, 2006 ▸) because of the unavailability of the components. The original authors’ solution did not correspond with either the cocrystal or the salt. The critical hydrogen atom was found in the middle of the short hydrogen bond [2.5395 (15) Å], with an O–H distance of 1.26 (2) Å and an N–H distance of 1.28 (2) Å. Calculations using the rSCAN, r2SCAN, PBE0 and PBE50 functionals consistently converged to give the salt. As the structure exhibited a short hydrogen bond, the DFT calculation on the level used was not reliable or suitable for cocrystal/salt distinguishing in this case.

#### 3.3.5. LEWRIO [2-Amino­pyrimidine (3,4-di­chloro­phen­oxy)­acetic acid]

We successfully prepared LEWRIO crystals (Lynch *et al.*, 1994 ▸). We refined the structure using the HAR method with the hydrogen atoms treated anisotropically. The structure appeared to be in a transition phase, with an N–H distance of 1.223 (19) Å and an O–H distance of 1.320 (19) Å. The disorder model refinement confirmed a transition phase with a hydrogen atom occupancy of 0.61 (3) : 0.39 (3) (salt state : cocrystal state). At the measurement temperature (180 K), the structure probably exists in the form of a disordered state between cocrystal and salt. The length of the experimental hydrogen bond in this structure was 2.540 (3) Å. We performed calculations using the rSCAN and r2SCAN functionals. The structure converged from both starting models into the salt for both functionals. Lastly, we used the PBE0 functional, which led to cocrystal from the cocrystal starting model and salt from the salt starting model. In this case, it was necessary to use the PBE0 functional to obtain a model consistent with the salt–cocrystal continuum model as obtained by HAR refinement.

#### 3.3.6. UJORAM [4,4′-Bi­pyridine bis­(2,4-di­nitro­phenol)]

We successfully prepared UJORAM crystals (Akutagawa *et al.*, 2003 ▸). The structure was refined using the HAR method, with the hydrogen atoms treated anisotropically. The hydrogen atom was found in the cocrystal position. Even the disorder model showed high hydrogen atom occupancy in the cocrystal state 0.23 (3) : 0.77 (3) (salt state : cocrystal state). The length of the experimental hydrogen bond in this structure was 2.542 (2) Å. We performed calculations using the rSCAN and r2SCAN functionals. The structure converged from both starting models into the salt for both functionals. Further calculations using the PBE0 functional resulted in convergence into the cocrystal from the cocrystal starting model and into salt from the salt starting model. In this case, we considered the DFT method on the given level unsuitable for obtaining reliable results.

#### 3.3.7. TIGNUT [(*E*)-4-(4-Methyl­styryl)pyridine (*E*)-but-2-enedioic acid]

We did not crystallise the TIGNUT structure (Bu *et al.*, 2007 ▸) because the data for the structure from the original publication were available. We tried to re-refine the original data. Due to the low number of parameters and the fact that the original data measurement was conducted at room temperature, it was not possible to treat hydrogen atoms anisotropically. After the HAR refinement, with the hydrogen atoms treated isotropically, the hydrogen atom appeared to be closer to the salt side, with an N–H distance of 1.20 (4) Å and an O–H distance of 1.35 (4) Å. The disorder model of the structure showed a hydrogen atom occupancy of 0.63 (9) : 0.37 (9) (salt state : cocrystal state). Bu *et al.* (2007 ▸) placed the hydrogen atoms based solely on geometry. Based on our reinterpretation of their data, we consider the structure at the measurement temperature (291 K) to probably exist in the form of a disordered state between cocrystal and salt forms. We performed calculations using the rSCAN and r2SCAN functionals. The results for the rSCAN and r2SCAN functionals were salt for both starting models. The PBE0 functional result led to two energetic minima: cocrystal from the cocrystal starting model and salt from the salt starting model. The presence of a short hydrogen bond [2.548 (3) Å] in this structure required the use of the PBE0 functional to detect the salt–cocrystal continuum characteristic of the phase.

#### 3.3.8. KIDNOB {2,6-Bis­[(imidazol-1-yl)methyl]-4-methyl­phenol hemikis(terephthalic acid)}

We did not attempt to crystallise the KIDNOB phase (Wang *et al.*, 2007 ▸) because of the unavailability of the components. We performed calculations using the rSCAN and r2SCAN functionals. The structure always converged into salt. We then used the PBE0 functional. After this calculation, the structure converged into cocrystal from the cocrystal starting model and salt from the salt starting model. As in the previous cases, the DFT method was not able to reproduce the experimental results due to the presence of a short hydrogen bond of 2.554 (5) Å. However, it is possible that the original authors overlooked the salt–cocrystal continuum characteristic of the phase, as detected by the PBE0 functional.

#### 3.3.9. UNEBOE [1,2,4,5-Benzenetetra­carb­oxy­lic acid bis­(1,7-phenanthroline)]

We managed to successfully prepare the UNEBOE crystals (Arora & Pedireddi, 2003 ▸). After the structure was refined using the HAR method, with hydrogen atoms treated anisotropically, the structure appeared to be in a transition phase, with an N–H distance of 1.27 (2) Å and an O–H distance of 1.28 (2) Å. The disorder model refinement showed a transition phase with a hydrogen atom occupancy of 0.53 (3) : 0.47 (3) (salt state : cocrystal state). At the temperature during the data collection, the structure probably exists in the form of a disordered state between cocrystal and salt form. The length of the experimental hydrogen bond in this structure was 2.562 (2) Å. We performed calculations using the rSCAN and r2SCAN functionals. The results for the rSCAN functional were salt for both starting models. The r2SCAN functional results led to two energetic minima: a cocrystal from the cocrystal starting model and a salt from the salt starting model; thus correctly detecting the experimentally confirmed state.

#### 3.3.10. JEDLAG [1,3-Bis­(3-pyridyl)­urea succinic acid]

We successfully prepared JEDLAG crystals (Reddy *et al.*, 2006 ▸). After the structure was refined using the HAR method, with the hydrogen atoms treated isotropically, the structure appeared to be a cocrystal. The hydrogen atoms in this structure were refined only isotropically because of the limited data quality. Refinement based on the disorder model showed a transition phase, with a hydrogen atom occupancy of 0.36 (5) : 0.64 (5) (salt state : cocrystal state). The length of the experimental hydrogen bond in this structure was 2.564 (3) Å. We performed calculations using the rSCAN and r2SCAN functionals. The results for the rSCAN functional were salt for both starting models. The r2SCAN functional results led to two energetic minima: a cocrystal from the cocrystal starting model and a salt from the salt starting model. As in the previous cases, the DFT method on the level used was not able to reproduce the experimental results due to the presence of a short hydrogen bond. However, it is possible that the original authors overlooked the salt–cocrystal continuum characteristic of the phase, as detected by the r2SCAN functional and the disorder model.

#### 3.3.11. OGEPIA [1,3-Di-4-pyridyl­propane 2-hy­droxy­benzene-1,4-di­carb­oxy­lic acid]

We did not try to crystallise the OGEPIA structure (Qin *et al.*, 2008 ▸) because the experimental data from the original publication were available. We reinterpreted the original data using HAR refinement with the hydrogen atoms treated isotropically. Due to the low number of parameters and the fact that the original data were collected at room temperature, it was not possible to treat hydrogen atoms anisotropically. After the refinement, the structure was clearly salt. The disorder model confirmed salt with a hydrogen atom occupancy of 0.87 (8) : 0.13 (8) (salt state : cocrystal state). We performed calculations using the rSCAN and r2SCAN functionals. The results for the rSCAN functional were salt from both starting models. The r2SCAN functional results led to two energetic minima: a cocrystal from the cocrystal starting model and a salt from salt starting model. Qin *et al.* (2008 ▸) placed the hydrogen atoms based solely on geometry. Based on our reinterpretation of their data, we believe the structure to be a salt. The presence of a short hydrogen bond [2.567 (4) Å] in this structure made the DFT method unreliable and unfit for distinguishing between salt and cocrystal. This case is an exception when rSCAN results are correct in comparison to the r2SCAN one.

#### 3.3.12. ODOHIZ [2-Hy­droxy­benzoic acid bis­(3,5-di­methyl-1H-pyrazole)]

We failed to prepare ODOHIZ crystals (López *et al.*, 2007 ▸). The length of the experimental hydrogen bond in this structure was 2.598 (5) Å. Calculations using the rSCAN, r2SCAN, PBE0 and PBE50 functionals consistently converged into salt. As the crystals could not be prepared, we checked the experimental settings of the original authors. There were many signs of poor quality of the data and the structural model. Data collection was performed at 298 K. While *R* converged to a reasonable value, the number of observed reflections [*I* > 2σ(*I*)] was only 41% of all reflections. There were 5.7 observed reflections per refined parameter (1253/221). The authors reported an *R*_int_ > 12% and a 10% decay during the data collection. Only one hydrogen atom (O⋯H⋯O intermolecular hydrogen bridge) was refined. None of the remaining hetero-bound hydrogen atoms found in the Fourier maps were refined. We can conclude that their refinement was not stable. The positions of the hydrogen atoms on the methyl groups were not optimized. Some of them even gave nonsense collision geometry. Based on the study of the experimental X-ray setup used for the determination of ODOHIZ by López *et al.* (2007 ▸), we believe that the hydrogen atom position was not determined correctly because the data were not suitable for the refinement of hydrogen atoms and the structure was really a salt.

#### 3.3.13. VODCOH [2,3,4,5-Tetra­kis­(4-pyridyl)­thio­phene benzene-1,2,4,5-tetra­carb­oxy­lic acid]

The VODCOH structure (Qiu *et al.*, 2008 ▸) could not be prepared and solved experimentally because of the cost of one component, 2,3,4,5-tetra­kis­(4-pyridyl)­thio­phene. For this structure, we performed geometry optimisations using the rSCAN, r2SCAN and PBE0 functionals. The structure always converged into salt. After the PBE50 functional calculation we detected the presence of two energetic minima: the structure converged into cocrystal from the cocrystal starting model and into salt from the salt starting model. As the crystals could not be prepared, we checked the experimental solution. The authors placed the hydrogen atoms based solely on geometry. Moreover, the length of the bonds in the carb­oxy­lic acid involved in the hydrogen atom transfer between oxygen and nitro­gen was suspicious: for the double bond C=O it was 1.249 (3) Å; for the single bond C—OH it was shorter, with a length of 1.239 (3) Å. We believe that the structure was not solved correctly as a cocrystal and that the DFT method detected a problematic structure. The PBE50 functional provided incorrect results in this case due to its cocrystal preference. The structure is salt with high probability, as the DFT method using the rSCAN functional should work reliably for the given type and length of hydrogen bond [2.618 (3) Å].

#### 3.3.14. MIPVOW [Bis(hexa­methyl­enetetra­amine) *m*-benzene­dicarb­oxy­lic acid]

MIPVOW crystals (Li *et al.*, 2001 ▸) were successfully prepared. After the anisotropic HAR refinement, the critical hydrogen atom was more on the cocrystal side, with distances of 1.229 (13) Å from oxygen and 1.363 (13) Å from nitro­gen. The disorder model showed a hydrogen atom occupancy of 0.365 (17) : 0.635 (17) (salt state : cocrystal state). The length of the experimental hydrogen bond in this structure was 2.626 (6) Å. Thus, the DFT method using the rSCAN functional should work reliably for the given type and length of the hydrogen bond. We performed calculations using the rSCAN, r2SCAN, PBE0 and PBE50 functionals from the original structure and false salt and the results were always salt. In the original publication, the authors placed the hydrogen atom in the middle of the distance between oxygen and nitro­gen (O3⋯H3⋯N1 1.32 Å and 1.33 Å) and did not mention how this hydrogen atom was detected. On the energetic surface, such a position could lead to movement to only one local minimum. Therefore, we decided to perform another calculation and create an artificial cocrystal in addition to the artificial salt. We then performed two calculations using the rSCAN functional — one for the artificial cocrystal, with a hydrogen atom bound to oxygen and an O—H distance of 0.95 Å, and another for the artificial salt, with a N—H distance of 0.95 Å. This approach led to two energetic minima: a cocrystal from the cocrystal starting model and a salt from the salt starting model. In this case, the DFT method correctly confirmed the originally detected salt–cocrystal continuum characteristic of the phase. It should be noted that this interesting situation was not discussed at all by the original authors.

#### 3.3.15. GIPQAX [Bis(maleic acid) 4,4′-bi­pyridine]

We successfully crystallised the GIPQAX structure (Chatterjee *et al.*, 1998 ▸). After the structure was refined using the HAR method, with the hydrogen atoms treated anisotropically, the structure was clearly salt. This is the same result as those found in two other publications that investigated the same structure (Bowes *et al.*, 2003 ▸; Lombard *et al.*, 2020 ▸). The disorder model confirmed salt with hydrogen atom occupancy of 0.920 (17) : 0.080 (17) (salt state : cocrystal state). We performed calculations using the rSCAN, r2SCAN and PBE0 functionals. The structure always converged into salt. The length of the experimental hydrogen bond in this structure was 2.630 (4) Å. For this length, the rSCAN functional should reliably predict the form of the crystal. After the PBE50 functional calculation we detected the presence of two energetic minima: the structure converged into cocrystal from the cocrystal starting model and into salt from the salt starting model. The DFT method correctly detected the structure incorrectly determined as a cocrystal. The PBE50 functional provided incorrect results due to its cocrystal preference in this case.

#### 3.3.16. CITSAZ10 (Imidazole 1,1′-bi­naphthyl-2,2′-di­carb­oxy­lic acid)

The CITSAZ10 structure (Czugler *et al.*, 1986 ▸) could not be prepared and solved experimentally because of the high cost of one component, 1,1′-bi­naphthyl-2,2′-di­carb­oxy­lic acid. The length of the experimental hydrogen bond in this structure was 2.773 (11) Å and the DFT method should reliably predict the form of the crystal. For this structure, we performed geometry optimisations using the rSCAN, r2SCAN, PBE0 and PBE50 functionals. The structure always converged into salt. However, the authors admitted that their experiment was poorly performed: the crystal was too small, it was not possible to perform anisotropic refinement even for heavy atoms, and the *R* factor was high. We believe that the structure was not solved correctly and that, in reality, it is salt.

## 4. Conclusions

We confirmed a correct cocrystal structure determination in 301 cases. For 87 structures, we have a dubious form determination, and the structures probably created a salt–cocrystal continuous form. The behaviour of this phase will be described in a separate study.

From 16 phases exhibiting consistent salt behaviour as determined by our methodology, we experimentally proved two true salts: OGEPIA, GIPQAX. For ODOHIZ, CITSAZ10 and VODCOH, we believe that the original authors did not solve the structures correctly and that the DFT method (rSCAN + MBD) correctly detected the issue. These three phases are salts as well.

We confirmed that, in some cases, the DFT method based only on the rSCAN functional was unreliable and unsuitable for distinguishing cocrystal/salt with a strong hydrogen bond. We also confirmed that advanced functionals (*i.e.* r2SCAN, PBE0 and PBE50) could be used in some cases to correct the rSCAN results. In two cases (*i.e.* GIPQAX and VODCOH), the PBE50 functional probably incorrectly preferred the existence of the cocrystal state and thus it is not suggested for future use. For VODCOH, MIPVOW, GIPQAX and CITSAZ10, which violated the reliability rule we had established in an earlier article (Hušák *et al.*, 2022 ▸), we confirmed that the DFT methodology based on the rSCAN functional worked correctly and that the problem was with the original experimental structure determination.

In five cases (*i.e.* GADGUN03, LEWRIO, TIGNUT, UNEBOE and MIPVOW), we experimentally found a form that appeared to be in a transition salt–cocrystal continuum phase. We believe that these structures at the measurement temperature (180 K/291 K for TIGNUT) probably exist in a form of a disordered state between cocrystal and salt form.

For future salt–cocrystal differentiation we suggest using the r2SCAN functional, which gives correct results for O—H⋯N bonds longer than 2.554 (5) Å [in contrast to our previous 2.613 (16) Å limit]. The computational costs of the r2SCAN functional are comparable with rSCAN, and its use for massive screening is more realistic than the use of computationally heavy hybrid functionals.

## Supplementary Material

Crystal structure: contains datablock(s) global, mo_GADGUN03_1_0m, mo_GIPQAX_0m, mo_JEDLAG_0m, mo_LEWRIO_0m, mo_MIPVOW_0m, mo_UJORAM_1_0ma, mo_UNEBOE_0m, OGEPIA, TIGNUT. DOI: 10.1107/S205252062500068X/dk5134sup1.cif

Supporting information file. DOI: 10.1107/S205252062500068X/dk5134sup2.pdf

Supporting information file. DOI: 10.1107/S205252062500068X/dk5134mo_GADGUN03_1_0msup3.cml

Supporting information file. DOI: 10.1107/S205252062500068X/dk5134mo_GIPQAX_0msup4.cml

Supporting information file. DOI: 10.1107/S205252062500068X/dk5134mo_JEDLAG_0msup5.cml

Supporting information file. DOI: 10.1107/S205252062500068X/dk5134mo_LEWRIO_0msup6.cml

Supporting information file. DOI: 10.1107/S205252062500068X/dk5134mo_UJORAM_1_0masup7.cml

CCDC references: 2420245, 2420246, 2420247, 2420248, 2420249, 2420250, 2420251, 2420252, 2420253

## Figures and Tables

**Table 1:** Summary of the structures processed during screening (rSCAN functional used)

	Cocrystal	Location of CSD codes
Structures found in Δp*K*_a_ interval 〈−1,4〉 according to Cruz-Cabeza (2012 ▸)	495	See supplementary data in Cruz-Cabeza (2012 ▸)
Structures selected for DFT calculation (no disorder, no critical errors in the structure)	404	Section S1.1
Structures converging to cocrystal from both starting models	301	Section S1.2
Structures converging to the local minima from both cocrystal and salt starting model	87	Section S1.3
Structures converting always to the salt model	16	Table 2 ▸; Section S1.4

**Table 2:** Results for problematic cocrystals converging during the main screening to salt from any model

CSD code	O⋯H⋯N distance (Å)	rSCAN + MBD result†	r2SCAN + MBD result†	PBE0+TS result†‡	PBE50 result†‡	Crystallisation / original experimental data available	HAR + H aniso /H iso refinement§	HAR + disorder modelling
LAPTUS	2.510 (2)	Salt / salt	Salt / salt	Cocr / salt	–	N	–	–
GADGUN03	2.519 (6)	Salt / salt	Salt / salt	Salt	Salt	Y	Salt–cocr continuum	NH 0.50 (5) OH 0.50 (5)
ULAWAF02	2.5294 (11)	Salt / salt	Cocr / salt	–	–	N	–	–
SEPLUV	2.5395 (15)	Salt / salt	Salt / salt	Salt	Salt	N	–	–
LEWRIO	2.540 (3)	Salt / salt	Salt / salt	Cocr / salt	–	Y	Salt–cocr continuum	NH 0.61 (3) OH 0.39 (3)
UJORAM	2.542 (2)	Salt / salt	Salt / salt	Cocr / salt	–	Y	Cocrystal	NH 0.23 (3) OH 0.77 (3
TIGNUT	2.548 (3)	Salt / salt	Salt / salt	Cocr / salt	–	Y	Salt–cocr continuum	NH 0.63 (9) OH 0.37 (9)
KIDNOB	2.554 (5)	Salt / salt	Salt / salt	Cocr / salt	–	N	–	–
UNEBOE	2.562 (2)	Salt / salt	Cocr / salt	–	–	Y	Salt–cocr continuum	NH 0.53 (3) OH 0.47 (3)
JEDLAG	2.564 (3)	Salt / salt	Cocr / salt	–	–	Y	Cocr	NH 0.36 (5) OH 0.64 (5)
OGEPIA	2.567 (4)	Salt / salt	Cocr / salt	–	–	Y	Salt	NH 0.87 (8) OH 0.13 (8)
ODOHIZ	2.598 (5)	Salt / salt	Salt / salt	Salt	Salt	N	–	–
VODCOH	2.618 (3)††	Salt / salt	Salt / salt	Salt	Cocr / salt	N	–	–
MIPVOW¶	2.626 (6)††	Salt / salt	Salt / salt	Salt	Salt	Y	Salt–cocr continuum	NH 0.365 (17) OH 0.635 (17)
		Cocr / salt						
GIPQAX	2.630 (4)††	Salt / salt	Salt / salt	Salt	Cocr / salt	Y	Salt	NH 0.920 (17) OH 0.080 (17)
CITSAZ10	2.773 (11)††	Salt / salt	Salt / salt	Salt	Salt	N	–	–

† DFT geometry optimisation result in the order of cocrystal starting model / artificial salt starting model.

‡ Due to the computer power consumption of the calculation using the PBE0 and PBE50 functionals and the limited computational sources, only one calculation was run for the case when the cocrystal starting model converged into salt. Only in the case when the cocrystal starting model converged to cocrystal, was the calculation from the salt starting model run.

§ Due to the low number of parameters it was not possible to treat hydrogen atoms anisotropically for structure TIGNUT (see Section 3.3.7), OGEPIA (see Section 3.3.11) and JEDLAG (see Section 3.3.10).

¶ See Section 3.3.14 for explanation of the results.

†† Bond distance violates the conclusion for the method reliability determined in our previous work.
