# Supplementary material for: Computation screening for incorrectly determined cocrystal structures
Source: Acta Crystallogr B Struct Sci Cryst Eng Mater. 2025 Feb 25;81(Pt 2):208–16. doi: 10.1107/S205252062500068X (PMC11970119; doi:10.1107/S205252062500068X)
Supplement: Supplementary file 2 [file b-81-00208-sup2.pdf]

STRUCTURAL SCIENCE
CRYSTAL ENGINEERING
MATERIALS

Volume 81 (2025)

Supporting information for article:

Computation screening for incorrectly determined cocrystal structures

Simona Chalupná, Michal Hušák, Jan Čejka, František Fňukal and Jiří Klimeš

S1. Refcodes of structures used for the screening

The complete results of the geometry optimization of the structures in CASTEP are available from the authors under request.

S1.1. Refcodes of all structures experimentally determined as cocrystals used in the study (404)

ADARIH, ADETOT, AJAKAX, AJAKEB, AJAKIF, AJEZEV, AJIWIA, ASAXOH, ASAXUN, BARMIM, BIJVOF, BIJVUL, BIJWOG, BIJYEY, BIZMAZ, BUDWAY10, BUDWEC, BUDZUV, BUFQAU, CAJQAG, CAJQEK, CAJQIO, CAJQOU, CAJRAH, CAJRIP, CIRXAD, CITSAZ10, COCMOX, COGREV, COWHUS, COWJAA, COWJOO, COZXIZ, COZXOF, COZXUL, COZYAS, COZYIA, COZYOG, COZYUM, CUJMIE, CUJMOK, CUJMUQ, CUJNAX, CUJNEB, CUMNEE, DATQOF, DAYWAC, DAYZOT, DEHSIS10, DEXTOQ, DIFNUC, DIFPAK, DIKCIK, DIKCOQ, DIKCUW, DIKDEH, DITHUK, DITJAS, DITJEW, DUCXOP, DUKVUB, EDOROE, EGUXAG, EJUZEO, EKECOM01, EMEBON, EYOJOR, FEQXIJ, FERYAD, FERYEH, FERYIL, FERYOR, FERYUX, FERZAE, FERZEI, FIHYEA, FIJCIK, FIJCUW, FUHGUL, GADGUN03, GAHDUP, GAHFAX, GAHFIF, GALBIF, GANXUP, GAWJEU, GAWLOG, GENNET, GENNIX, GEYSAE, GIMPAU, GIPQAX, GIPQEB, GISGUK, GOBYOL, GOBZEC, GODNAO, GODQIZ, GOJQAY, GUHTUZ, GUKWOZ, GUYBUX, HANBOO, HEQZOT, HIHPEU, HONTAG, HONTOU, HONTUA, HONVIQ, HONVOW, HONVUC, HONWAJ, HOZBOO, HUHQUW, HUHRAD, HUSTOE, HUSVIA, HUSWIB, IFACUO, IFUQAC, IGABEY, IXOHAE, IYUNOF, JAQNOF, JAWVOT, JAWWAG, JAZBES, JEDLAG, JEGBON, JESMOK, JIQCAN, KATBAJ, KAVDUH, KEFBAZ, KEFBED, KEFBIH, KEFBUT, KEFCAA, KEFCEE, KEGCIJ, KEKGAJ, KEKGUD, KEKHIS, KIDNOB, KIDNUH, KIXVES, KRLAPY10, KURGOU, LAPTUS, LAPVAA, LATKUN, LATLAU, LATLEY, LATLIC, LATLOI, LAWBER, LEWREK, LEWRIO, LIPXEN, LIQTIP, LOLSUA, LUDFUL, LUJBUO, LUNMAI, LUNMEM, LUNMIQ, LUNMOW, LUNMUC, LUNNAJ, LUNNEN, LUNNIR, LUNNOX, LUNNUD, LUNPAL, LUNPEP, MAHWUO, MEBRAN, MEHFOV, MELYEI, MIPVIQ, MIPVOW, MIZQAO, MODPAX, MOVTOH, MOXTOJ, MUPQET, NANQAV, NANQID, NANQOJ, NEWRIR, NEWROX, NEWRUD, NEXXIX, NIFCAH, NIQNIL, NIXLUC, NOKWUF, NOSZAW, NOTKIQ, NOVSOG, NOVTAT, NUGQOW, NUGVUH, NUGZEV, NUGZEV01, NUGZOF, NUHBAU, NUKWEW, NUKWEW01, NUKWOG, NUKXAT, NUQKOA, OCAYUM, OCAZAT, ODOHIZ, OFOKEA, OFOKOK, OGEPIA, OJENIA, PAPFOB, PAXNOR, PEGDAH, PEQPAD, PEQPIL, PEQPOR, PEQQAE, PEQQIM, PEQQOS, POFVOV, PUJHUY, PUJNIS, PUKDIJ, QAWTUE, QIPKOP, QOLSAM,

QUFYOG, QUFZEX, QUJDOP, QUJDOP01, QUJFIL, RADFEI, RADGAF, RAHHEN, RAPHEV01, RAPRIK, REFNIA, RESFOL, RESGAY, RESGIG, RESGOM, RESHAZ, RETZEW, RIHBUG, RIZWOM, ROLFUU, RONDAA, RONDEE, RONDII, RUJCII, RUJRUK, RUXPUV, SASBEU, SASBIY, SAYMUB, SAYNIQ, SAYNOW, SAYNUC, SAYPAK, SAYPEO, SAYPOY, SAYPUE, SAYQAL, SEPLUV, SEPSIP, SEPSIP01, SERMOR, SIQBIE, SIYCEJ, SIYCIN, SIYWAZ, SODCEU, SODKIG, SODKOM, SODKUS, SOGXUH, SOMKEL, SOVFOY, SUVJEY, SUXVUC, TAZWOH, TEFSON, TEJYEN, TETXUL, TEVWOH, TIDFER, TIDFUH, TIFSUW, TIFTAD, TIGNUT, TIJKOM, TIPFII, TITHAG, TOJCEB, TONMAL, TONMEP, UCEXAB, UCEXUV, UDUZEY, UDUZOI, UHAPUO, UHAQAV, UHAQEZ, UHAQID, UHAQOJ, UHAQUP, UHUVEZ, UJORAM, UJOREQ, ULAWAF, ULAWAF02, ULAWEJ, ULAWOT, ULAWUZ, ULAXAG, ULAXEK, UMIMOS, UMIMUY, UMINAF, UMINEJ, UMININ, UMINOT, UNEBOE, UNECAR, VAKTOR, VAPFEX, VEFVIM, VIJTIR, VOCZET, VODCOH, VOJHAE, VORCOV, VUGMIT, VUYVIV, VUZCIC, VUZCOI, WANCOE, WANCUK, WANDIZ, WAPXOA, WARPIP, WEDLUN, WEPBID, WEPDIF, WEVKAK, WINMIQ, WOPZUW, WOQBAF, WOQBEJ, WOQBIN, WUKRAV, WUKREZ, WUKROJ, XAPMAC, XAPMIK, XAPWOA, XAQPEL, XAQPOV, XAQQEM, XEBFOA, XEXCAE, XEZDIQ, XIFSUB, XIPTOG, XIPVAU, XOHWEX, XOJMUF, XOJNAM, XOJNIU, XOJNOA, XOJNUG, XONPOF, XUNHAP, YAKVAI, YAKVEM, YAKVIQ, YAMPIM, YECJAS, YECHAQ, YECHIY, YECHOE, YECHUK, YEJKON, YIRLOB, YOCSIT, YUCVAU, YUQYOZ, YUQYUF, ZAJHOH, ZAJJEZ, ZINZUR, ZIPBEF, ZUDBIJ, ZUPKUQ, ZUPLAX

S1.2. Refcodes of experimentally determined cocrystals converging to cocrystal from both starting models (301)

ADARIH, AJAKAX, AJAKIF, AJEZEV, ASAXOH, ASAXUN, BARMIM, BIJVOF, BIJVUL, BIJWOG, BIJYEY, BIZMAZ, BUDWAY10, BUDWEC, CAJQEK, CAJQOU, CAJRAH, CAJRIP, CIRXAD, COCMOX, COGREV, COWJOO, COZXIZ, COZXOF, COZXUL, COZYAS, COZYIA, COZYOG, COZYUM, CUJMIE, CUJMOK, CUJMUQ, CUJNAX, CUJNEB, CUMNEE, DATQOF, DAYWAC, DAYZOT, DEHSIS10, DEXTOQ, DIFNUC, DIFPAK, DIKCIK, DIKCUW, DIKDEH, DITHUK, DITJAS, DITJEW, DUKVUB, EDOROE, EGUXAG, EJUZEO, EKECOM01, EYOJOR, FEQXIJ, FERYAD, FERYEH, FERYIL, FERYOR, FERYUX, FERZAE, FERZEI, FIHYEA, FIJCIK, FIJCUW, GAHDUP, GAHFAX, GAWJEU, GAWLOG, GENNET, GENNIX, GIMPAU, GIPQEB, GISGUK, GOBYOL, GOBZEC, GODQIZ, GOJQAY, GUHTUZ, GUKWOZ, GUYBUX, HANBOO, HIHPEU, HONTAG, HONTOU, HONTUA, HONVIQ, HONVOW, HONVUC, HONWAJ, HOZBOO, HUHQUW, HUHRAD, HUSTOE, HUSVIA, HUSWIB, IFUQAC, IGABEY, IXOHAE, JAQNOF, JAWWAG, JAZBES, JEGBON, JESMOK, JIQCAN, KATBAJ, KEFBIH, KEFBUT, KEFCAA, KEFCEE, KEGCIJ, KEKHIS, KIDNUH, KIXVES, KRLAPY10, LATKUN, LATLAU, LATLEY, LATLIC, LATLOI, LAWBER, LEWREK, LIPXEN, LIQTIP, LUJBUO,

LUNMAI, LUNMEM, LUNMIQ, LUNMUC, LUNNEN, LUNNIR, LUNNOX, LUNNUD, LUNPAL, LUNPEP, MAHWUO, MEBRAN, MEHFOV, MIPVIQ, MIZQAO, MOVTOH, MOXTOJ, MUPQET, NANQAV, NANQID, NANQOJ, NEWRIR, NEWROX, NIXLUC, NOKWUF, NOSZAW, NOTKIQ, NOVSOG, NOVTAT, NUGQOW, NUGVUH, NUGZEV, NUGZEV01, NUKWEW, NUKWEW01, NUKXAT, NUQKOA, OCAYUM, OCAZAT, OFOKOK, OJENIA, PAPFOB, PEGDAH, POFVOV, PUJHUY, QOLSAM, QUFYOG, QUFZEX, QUJDOP, QUJDOP01, QUJFIL, RADFEI, RAHHEN, RAPHEV01, RAPRIK, REFNIA, RESGAY, RESGIG, RESGOM, RESHAZ, RETZEW, RIHBUG, RIZWOM, ROLFUU, RONDAA, RONDEE, RONDII, RUJCII, RUJRUK, RUXPUV, SASBEU, SASBIY, SAYNIQ, SAYNOW, SAYNUC, SAYPAK, SAYPEO, SAYPOY, SAYPUE, SAYQAL, SEPSIP, SEPSIP01, SERMOR, SIQBIE, SIYWAZ, SODCEU, SODKIG, SODKOM, SODKUS, SOMKEL, SOVFOY, SUVJEY, SUXVUC, TAZWOH, TEFSON, TEJYEN, TEVWOH, TIDFER, TIFSUW, TIFTAD, TITHAG, TOJCEB, TONMAL, UCEXAB, UCEXUV, UDUZEY, UDUZOI, UHAPUO, UHAQAV, UHAQEZ, UHAQID, UHAQUP, UHUVEZ, UJOREQ, ULAWOT, ULAWUZ, ULAXAG, ULAXEK, UMIMOS, UMIMUY, UMINEJ, UMININ, UMINOT, UNECAR, VAKTOR, VAPFEX, VEFVIM, VIJTIR, VOJHAE, VUZCIC, VUZCOI, WANCUK, WAPXOA, WARPIP, WEDLUN, WEVKAK, WINMIQ, WOPZUW, WOQBAF, WOQBEJ, WOQBIN, WUKRAV, WUKREZ, WUKROJ, XAPMIK, XAPWOA, XAQPEL, XAQQEM, XEBFOA, XEXCAE, XIPTOG, XIPVAU, XOHWEX, XOJNAM, XOJNIU, XOJNOA, XOJNUG, XUNHAP, YAKVAI, YAKVEM, YAKVIQ, YAMPIM, YECJAS, YECHAQ, YECHIY, YECHOE, YECHUK, YEJKON, YIRLOB, YOCSIT, ZAJHOH, ZAJJEZ, ZINZUR, ZIPBEF, ZUDBIJ, ZUPLAX

S1.3. Refcodes of experimentally determined cocrystals converging to salt from salt starting model and cocrystal from cocrystal starting model (87)

ADETOT, AJAKEB, AJIWIA, BUDZUV, BUFQAU, CAJQAG, CAJQIO, COWHUS, COWJAA, DIKCOQ, DUCXOP, EMEBON, FUHGUL, GAHFIF, GALBIF, GANXUP, GEYSAE, GODNAO, HEQZOT, IFACUO, IYUNOF, JAWVOT, KAVDUH, KEFBAZ, KEFBED, KEKGAJ, KEKGUD, KURGOU, LAPVAA, LOLSUA, LUDFUL, LUNMOW, LUNNAJ, MELYEI, MODPAX, NEWRUD, NEXXIX, NIFCAH, NIQNIL, NUGZOF, NUHBAU, NUKWOG, OFOKEA, PAXNOR, PEQPAD, PEQPIL, PEQPOR, PEQQAE, PEQQIM, PEQQOS, PUJNIS, PUKDIJ, QAWTUE, QIPKOP, RADGAF, RESFOL, SAYMUB, SIYCEJ, SIYCIN, SOGXUH, TETXUL, TIDFUH, TIJKOM, TIPFII, TONMEP, UHAQOJ, ULAWAF, ULAWEJ, UMINAF, VOCZET, VORCOV, VUGMIT, VUYVIV, WANCOE, WANDIZ, WEPBID, WEPDIF, XAPMAC, XAQPOV, XEZDIQ, XIFSUB, XOJMUF, XONPOF, YUCVAU, YUQYOZ, YUQYUF, ZUPKUQ

S1.4. Refcodes of experimentally determined cocrystals converging to salt from both starting models (16)

CITSAZ10, GADGUN03, GIPQAX, JEDLAG, KIDNOB, LAPTUS, LEWRIO, MIPVOW, ODOHIZ, OGEPIA, SEPLUV, TIGNUT, UJORAM, ULAWAF02, UNEBOE, VODCOH

S2. Detailed parameters of the DFT calculations setup

S2.1. rSCAN functional

Table S1 DFT calculation setup used for rSCAN functional calculations.

Parameter	Value	Units
CASTEP version	22.11	
functional	rSCAN	
Dispersion correction	MBD	
Pseudopotential used	ultrasoft, on the fly	
Energy cut-off	625.8619	eV
total energy convergence tolerance	1.00e-06	eV/atom
max ionic/ force tolerance	0.03	eV/Å
max ionic /displacement tolerance	0.001	Å

S2.2. r2SCAN functional

Table S2 DFT calculation setup used for r2SCAN functional calculations.

Parameter	Value	Units
CASTEP version	23.11	
functional	r2SCAN	
Dispersion correction	MBD	
Pseudopotential used	ultrasoft, on the fly	
Energy cut-off	625.8619	eV
total energy convergence tolerance	1.00e-06	eV/atom
max ionic/ force tolerance	0.03	eV/Å
max ionic /displacement tolerance	0.001	Å

S2.3. PBE0 functional

Table S3 DFT calculation setup used for PBE0 functional calculations.

Parameter	Value	Units
CASTEP version	23.11	
functional	PBE0	
Dispersion correction	Tkatchenko-Scheffler	
Pseudopotential used	norm-conserving, on the fly	
Energy cut-off	707.4960	eV
total energy convergence tolerance	2.00e-06	eV/atom
max ionic/ force tolerance	0.05	eV/Å
max ionic /displacement tolerance	0.002	Å

S2.4. PBE50 functional

Table S4 DFT calculation setup used for PBE50 functional calculations.

Parameter	Value	Units
CASTEP version	23.11	
functional	PBE50 (defined as custom XC: HF 0.5 PBE_X 0.5 PBE_C 1.0)	
Dispersion correction	MBD	
Pseudopotential used	norm-conserving, on the fly	
Energy cut-off	707.4960	eV
total energy convergence tolerance	2.00e-06	eV/atom
max ionic/ force tolerance	0.05	eV/Å
max ionic /displacement tolerance	0.002	Å

S3. Crystallisation and data measurement

S3.1. GADGUN03

100 mg of pentachlorophenol was dissolved in 8 ml of Freon 112 while heating. Subsequently, 100 µl of 4-methylpyridine was added. The sample was left to crystallise at room temperature and the crystals formed after a few days.

Table S5 Crystallographic information GADGUN03

**4-Methylpyridine pentachlorophenol**	
**Crystal data**	
Chemical formula	$C_{12}H_8Cl_5NO$
M_r	359.47
Crystal system, space group	Triclinic, *P*-1
Temperature (K)	180
a, *b*, *c* (Å)	7.3317 (3), 8.9118 (3), 11.8169 (4)
α, β, γ (°)	69.929 (1), 84.854 (1), 76.227 (1)
V (Å^3)	704.33 (5)
Z	2
Radiation type	Mo *K*α
μ (mm^{-1})	1.02
Crystal size (mm)	0.31 × 0.26 × 0.17
**Data collection**	
Diffractometer	Bruker D8 Venture
Absorption correction	Multi-scan<br>Data were corrected for absorption effects using the Multi-Scan method (*SADABS*)
T_{min}, T_{max}	0.652, 0.747
No. of measured, independent and observed [$I \geq 2u(I)$] reflections	145175, 4674, 4118
R_{int}	0.041
(sin θ/λ)$_{max}$ (Å^{-1})	0.735
**Refinement**	
$R[F^2 > 2\sigma(F^2)]$, $wR(F^2)$, S	0.023, 0.089, 1.00
No. of reflections	4674
No. of parameters	244
H-atom treatment	All H-atom parameters refined
$\Delta\rangle_{max}$, $\Delta\rangle_{min}$ (e Å^{-3})	0.34, -0.24
Computer programs:	*SHELXT* 2018/2 (Sheldrick, 2018), olex2.refine 1.5 (Bourhis *et al.*, 2015), Olex2 1.5 (Dolomanov *et al.*, 2009).

S3.2. GIPQAX

4,4'-bipyridine (156 mg, 1 mmol) was dissolved in methanol (30 ml). Maleic acid (232 mg, 2 mmol) was added, dissolved by heating to about 40° C for 10 minutes, and the solution was allowed to slowly evaporate in open vial. Single crystals of 4,4'-bipyridine - maleic acid (1:2) were obtained within 3 days.

Table S6 Crystallographic information GIPQAX

**Bis(Maleic acid) 4,4'-bipyridine**	
**Crystal data**	
Chemical formula	$2(C_4H_3O_4)\cdot C_{10}H_{10}N_2$
M_r	388.34
Crystal system, space group	Monoclinic, *C*2/*c*
Temperature (K)	180
a, *b*, *c* (Å)	23.9983 (9), 6.7578 (3), 11.4431 (4)
β (°)	116.0336 (11)
V (Å^3)	1667.50 (11)
Z	4
Radiation type	Mo *K*a
m (mm^{-1})	0.12
Crystal size (mm)	0.52 × 0.35 × 0.22
**Data collection**	
Diffractometer	Bruker D8 Venture
Absorption correction	Multi-scan Data were corrected for absorption effects using the Multi-Scan method (SADABS).
T_{min}, T_{max}	0.703, 0.749
No. of measured, independent and observed [$I \geq 2u(I)$] reflections	213706, 4947, 4621
R_{int}	0.035
(sin q/l)$_{max}$ (Å^{-1})	0,893
**Refinement**	
$R[F^2 > 2s(F^2)]$, $wR(F^2)$, S	0.017, 0.039, 1.09
No. of reflections	4947
No. of parameters	199
H-atom treatment	All H-atom parameters refined
Δ〉$_{max}$, Δ〉$_{min}$ (e Å^{-3})	0.25, -0.23
Computer programs:	olex2.solve 1.5 (Bourhis *et al.*, 2015), olex2.refine 1.5 (Bourhis *et al.*, 2015), Olex2 1.5 (Dolomanov *et al.*, 2009).

S3.3. JEDLAG

The synthesis of the component N,N´-bis(3-pyridyl)urea was carried out according to the original publication: 940 mg of 3-aminopyridine and 300 mg of urea were put into a vial, the sample was placed in an oven at 160° for 5 hours. After cooling, water was added to the vial (the product is not soluble in it), then the solid was centrifuged from the liquid phase and as much water as possible was pipetted off. The sample was then purged with EtOAc, 35 ml EtOAc was added and heated until the solid product dissolved - the sample dissolved and was allowed to recrystallise overnight. The next day, we washed the product first with EtOAc and hexane solution on the frit, then washed the solution with petroleum ether on the frit. 40 mg of product and 24 mg of succinic acid were dissolved in 2 ml of ethanol. The sample was left to crystallise at room temperature and crystals formed after several weeks.

Table S7 Crystallographic information JEDLAG

**1,3-bis(3-Pyridyl)urea succinic acid**	
**Crystal data**	
Chemical formula	$C_{15}H_{16}N_4O_5$
M_r	332.32
Crystal system, space group	Monoclinic, *C*2/*c*
Temperature (K)	180
a, *b*, *c* (Å)	37.809 (13), 5.2412 (19), 16.254 (6)
β (°)	108.433 (18)
V (Å^3)	3055.7 (19)
Z	8
Radiation type	Mo *K*α
μ (mm^{-1})	0.11
Crystal size (mm)	0.31 × 0.25 × 0.07
**Data collection**	
Diffractometer	Bruker D8 Venture
Absorption correction	Multi-scan<br>Data were corrected for absorption effects using the Multi-Scan method (*SADABS*)
T_{min}, T_{max}	0.7028, 0.7474
No. of measured, independent and observed [$I \geq 2u(I)$] reflections	204278, 3173, 2882
R_{int}	0.041
$(\sin \theta/\lambda)_{max}$ (Å^{-1})	0.628
**Refinement**	
$R[F^2 > 2\sigma(F^2)]$, $wR(F^2)$, S	0.045, 0.098, 1.08
No. of reflections	3167
No. of parameters	280

H-atom treatment	H atoms treated by a mixture of independent and constrained refinement
$\Delta\rangle_{max}$, $\Delta\rangle_{min}$ (e Å^{-3})	0.28, -0.22
Computer programs	olex2.refine 1.5 (Bourhis et al., 2015), Olex2 1.5 (Dolomanov et al., 2009).

S3.4. LEWRIO

100 mg phenoxyacetic acid and 43.0239 mg aminopyrimidine were dissolved together in 5 ml ethanol. The sample was then heated for 20 min. Crystals were obtained after a few days of slow evaporation.

Table S8 Crystallographic information LEWRIO

**2-Aminopyrimidine (3,4-dichlorophenoxy)acetic acid**	
**Crystal data**	
Chemical formula	$C_{12}H_{11}Cl_2N_3O_3$
M_r	316.15
Crystal system, space group	Triclinic, *P*-1
Temperature (K)	180
a, *b*, *c* (Å)	6.3868 (2), 6.9694 (3), 15.2424 (6)
α, β, γ (°)	92.0313 (14), 93.0261 (13), 103.6005 (13)
V (Å^3)	657.73 (4)
Z	2
Radiation type	Mo *K*a
m (mm^{-1})	0.50
Crystal size (mm)	0.32 × 0.21 × 0.13
**Data collection**	
Diffractometer	Bruker D8 Venture
Absorption correction	Multi-scan<br>Data were corrected for absorption effects using the Multi-Scan method (*SADABS*).
T_{min}, T_{max}	0.6872, 0.7479
No. of measured, independent and observed [$I \geq 2u(I)$] reflections	165782, 5778, 5281
R_{int}	0.035
(sin q/l)$_{max}$ (Å^{-1})	0.806
**Refinement**	
$R[F^2 > 2s(F^2)]$, $wR(F^2)$, S	0.023, 0.053, 1.11
No. of reflections	5778
No. of parameters	280
H-atom treatment	All H-atom parameters refined
$\Delta\rangle_{max}$, $\Delta\rangle_{min}$ (e Å^{-3})	0.38, -0.19
Computer programs	olex2.refine 1.5 (Bourhis et al., 2015), Olex2 1.5 (Dolomanov et al., 2009).

S3.5. MIPVOW

1,3-benzenedicarboxylic acid (166 mg, 1 mmol) was dissolved in ethanol (20 ml). Hexamethylenetetramine (280mg, 2 mmol) was added, dissolved by heating to about 60 C, 10 minutes and adding few water drops. The vial with thesolution was placed in diethyl ether vapours and single crystals of isophthalic acid - hexamethylenetetramine (1:2) were formed within 5 days.

Table S9 Crystallographic information MIPVOW

**Bis(Hexamethylenetetraamine) m-benzenedicarboxylic acid**	
**Crystal data**	
Chemical formula	$C_{14}H_{18}N_4O_4 \cdot C_6H_{12}N_4$
M_r	446.51
Crystal system, space group	Orthorhombic, $P2_12_12_1$
Temperature (K)	180
a, *b*, *c* (Å)	6.9874 (2), 13.8458 (4), 21.4834 (6)
V (Å^3)	2078.44 (10)
Z	4
Radiation type	Mo $K\alpha$
μ (mm^{-1})	0.10
Crystal size (mm)	0.534 x 0.221 x 0.133
**Data collection**	
Diffractometer	Bruker D8 Venture
Absorption correction	Multi-scan<br>Data were corrected for absorption effects using the Multi-Scan method (*SADABS*).
T_{min}, T_{max}	0.6872, 0.7488
No. of measured, independent and observed [$I \geq 2u(I)$] reflections	365157, 6037, 5934
R_{int}	0.053
$(\sin \theta/\lambda)_{max}$ (Å^{-1})	0.703
**Refinement**	
$R[F^2 > 2\sigma(F^2)]$, $wR(F^2)$, S	0.012, 0.029, 1.13
No. of reflections	6033
No. of parameters	559
H-atom treatment	All H-atom parameters refined
$\Delta\rangle_{max}$, $\Delta\rangle_{min}$ (e Å^{-3})	0.14, -0.08

Computer programs	olex2.refine 1.5 (Bourhis et al., 2015), Olex2 1.5 (Dolomanov et al., 2009).

S3.6. OGEPIA

Table S10 Crystallographic information OGEPIA

**1,3-Di-4-pyridylpropane 2-hydroxybenzene-1,4-dicarboxylic acid**	
**Crystal data**	
Chemical formula	$C_{29}H_{26}N_2O_{10}$
M_r	562.54
Crystal system, space group	Monoclinic, *C*2/*c*
Temperature (K)	291
a, *b*, *c* (Å)	22.9392 (11), 4.7809 (2), 24.1632 (11)
β (°)	96.542 (6)
V ($Å^3$)	2632.72 (1)
Z	4
Radiation type	Mo *K*α
μ (mm^{-1})	0.11
Crystal size (mm)	0.35 × 0.19 × 0.05
**Data collection**	
Diffractometer	Bruker *SMART* CCD area-detector
Absorption correction	Multi-scan<br>(*SADABS*; Bruker, 1997)
T_{min}, T_{max}	0.963, 0.994
No. of measured, independent and observed [$I \geq 2u(I)$] reflections	2444, 2444, 1335
R_{int}	0.049
$(\sin \theta/\lambda)_{max}$ ($Å^{-1}$)	0.606
**Refinement**	
$R[F^2 > 2\sigma(F^2)]$, $wR(F^2)$, S	0.047, 0.129, 1.02
No. of reflections	2444
No. of parameters	237
H-atom treatment	H atoms treated by a mixture of independent and constrained refinement
$\Delta\rangle_{max}$, $\Delta\rangle_{min}$ (e $Å^{-3}$)	0.39, -0.30
Computer programs:	*SMART* (Bruker, 1997), *SAINT* (Bruker, 1997), *SHELXS97* (Sheldrick, 2008), olex2.refine 1.5 (Bourhis *et al.*, 2015), Olex2 1.5 (Dolomanov *et al.*, 2009).

S3.7. TIGNUT

Table S11 Crystallographic information TIGNUT

**(E)-4-(4-Methylstyryl)pyridine (E)-but-2-enedioic acid**	
**Crystal data**	
Chemical formula	$C_{14}H_{14}N{\cdot}C_{14}H_{13}N{\cdot}C_4H_3O_4$
M_r	506.61
Crystal system, space group	Monoclinic, *Cc*
Temperature (K)	291
a, *b*, *c* (Å)	21.952 (10), 7.3477 (3), 17.0000 (7)
β (°)	104.610 (2)
V (Å^3)	2653.38 (1)
Z	4
Radiation type	Mo *K*α
μ (mm^{-1})	0.08
Crystal size (mm)	0.25 × 0.24 × 0.23
**Data collection**	
Diffractometer	Rigaku R-AXIS RAPID
Absorption correction	Multi-scan (*ABSCOR*; Higashi, 1995)
T_{min}, T_{max}	0.979, 0.981
No. of measured, independent and observed [$I \geq 2u(I)$] reflections	3033, 3032, 2447
R_{int}	0.026
$(\sin \theta/\lambda)_{max}$ (Å^{-1})	0.649
**Refinement**	
$R[F^2 > 2\sigma(F^2)]$, $wR(F^2)$, S	0.042, 0.101, 0.98
No. of reflections	3032
No. of parameters	463
H-atom treatment	All H-atom parameters refined
$\Delta\rangle_{max}$, $\Delta\rangle_{min}$ (e Å^{-3})	0.41, -0.30
Computer programs:	*SMART* (Bruker, 1997), *SAINT* (Bruker, 1997), *SHELXS97* (Sheldrick, 2008), olex2.refine 1.5 (Bourhis *et al.*, 2015), Olex2 1.5 (Dolomanov *et al.*, 2009).

S3.8. UJORAM

25 mg of BPY 4,4-bipyridine and 59 mg of DP 2,4-dinitrophenol were dissolved in 12 ml of ethanol and 4 ml of water and the sample was heated to dissolution. The sample was left to crystallise at room temperature and crystals formed after several weeks.

Table S12 Crystallographic information UJORAM

**4,4'-Bipyridine bis(2,4-dinitrophenol)**	
**Crystal data**	
Chemical formula	$C_{22}H_{16}N_6O_{10}$
M_r	524.41
Crystal system, space group	Orthorhombic, *Pbcn*
Temperature (K)	180
a, *b*, *c* (Å)	14.9666 (9), 7.0415 (4), 21.8350 (13)
V (Å^3)	2301.1 (2)
Z	4
Radiation type	Mo *K*α
μ (mm^{-1})	0.12
Crystal size (mm)	0.32 × 0.11 × 0.08
**Data collection**	
Diffractometer	Bruker D8 Venture
Absorption correction	Multi-scan<br>Data were corrected for absorption effects using the Multi-Scan method (*SADABS*).
T_{min}, T_{max}	0.7082, 0.7474
No. of measured, independent and observed [$I \geq 2u(I)$] reflections	263766, 2021, 1901
R_{int}	0.055
$(\sin \theta/\lambda)_{max}$ (Å^{-1})	0.595
**Refinement**	
$R[F^2 > 2\sigma(F^2)]$, $wR(F^2)$, S	0.022, 0.057, 1.04

No. of reflections	2021
No. of parameters	244
H-atom treatment	All H-atom parameters refined
$\Delta\rangle_{max}$, $\Delta\rangle_{min}$ (e Å^{-3})	0.43, -0.29
Computer programs	olex2.refine 1.5 (Bourhis et al., 2015), Olex2 1.5 (Dolomanov et al., 2009).

S3.9. UNEBOE

30 mg of 1,7-phenatroline and 42 mg of 1,2,4,5-benzenetetracarboxylic acid were individually dissolved in 1 ml of EtOH, mixed, and then an additional 10 ml of EtOH was added until the mixture dissolved. Crystals were obtained after a few days of slow evaporation.

Table S13 Crystallographic information UNEBOE

**1,2,4,5-Benzene tetracarboxylic acid bis(1,7-phenanthroline**	
**Crystal data**	
Chemical formula	$C_{33.982}H_{21.862}N_4O_{7.927}$
M_r	613.07
Crystal system, space group	Triclinic, *P*-1
Temperature (K)	180
a, *b*, *c* (Å)	7.0595 (7), 7.2153 (7), 13.9211 (13)
α, β, γ (°)	91.067 (4), 102.473 (4), 106.000 (4)
V ($Å^3$)	663.22 (11)
Z	1
Radiation type	Mo *K*α
μ (mm^{-1})	0.11
Crystal size (mm)	0.57 × 0.10 × 0.08
**Data collection**	
Diffractometer	Bruker D8 Venture
Absorption correction	Multi-scan<br>Data were corrected for absorption effects using the Multi-Scan method (*SADABS*).
T_{min}, T_{max}	0.7151, 0.7474
No. of measured, independent and observed [$I \geq 2u(I)$] reflections	86653, 3045, 2678
R_{int}	0.051
$(\sin \theta/\lambda)_{max}$ ($Å^{-1}$)	0.649
**Refinement**	
$R[F^2 > 2\sigma(F^2)]$, $wR(F^2)$, S	0.030, 0.058, 1.11
No. of reflections	3045
No. of parameters	307
H-atom treatment	All H-atom parameters refined
$\Delta\rangle_{max}$, $\Delta\rangle_{min}$ (e $Å^{-3}$)	0.16, -0.14
Computer programs	olex2.refine 1.5 (Bourhis et al., 2015), Olex2 1.5 (Dolomanov et al., 2009).

S3.10. Single-crystal X-ray diffraction and refinement

The structural data from monocrystals were collected using Bruker D8 VENTURE system equipped with a Photon II 7 CPAD detector, a multilayer monochromator, and a Mo Kα1 (λ = 0.71073 Å) sealed tube was used to collect all the intensity data. Crystal structures were determined at 180 K. The data reduction and absorption correction were performed with Apex3 software. To get the best possible hydrogen atom position, we had used for refinement the HAR method as implemented in Olex2 software and NoSpherA2 module (Jayatilaka & Dittrich 2008, Capelli et al. 2014, Kleemiss et al. 2020, Midgley et al. 2021).

Table S14 HAR refinement

Parameter	Value
Software	Olex2 + NoSperA2 module
Basis set	Def2-TZVP
Method	R2SCAN, Orca 5.0 code
H treatment	H anisotropic refinement / H isotropic refinement (JEDLAG, OGEPIA, TIGNUT)

Table S15 Modelling disorder + HAR refinement

Parameter	Value
Software	Olex2 + NoSperA2 module
Basis set	Def2-TZVP
Method	R2SCAN, Orca 5.0 code
Dfix restraints on N–H and O–H distance	0.95 (0.01)
Disordered H isotropic thermal parameter constrain	1.2 x U_{iso} of neighboring atom
H treatment	H mixed anisotropic refinement / H isotropic refinement (TIGNUT, JEDLAG)
